# Qualitative and Quantitative Analysis of Contrast-Enhanced Ultrasound in the Characterization of Kidney Cancer Subtypes

**DOI:** 10.3390/diagnostics15141795

**Published:** 2025-07-16

**Authors:** Daniel Vas, Blanca Paño, Alexandre Soler-Perromat, Daniel Corominas, Rafael Salvador, Carmen Sebastià, Laura Buñesch, Carlos Nicolau

**Affiliations:** Department of Radiology, Hospital Clínic de Barcelona, 08036 Barcelona, Spain

**Keywords:** contrast-enhanced ultrasound (CEUS), renal cell carcinoma (RCC), qualitative imaging analysis, quantitative perfusion analysis, clear cell RCC, papillary RCC, chromophobe RCC

## Abstract

**Objectives**: The aim of the study was to assess the utility of contrast-enhanced ultrasound (CEUS), using both qualitative and quantitative perfusion analysis, in differentiating subtypes of renal cell carcinoma (RCC). **Methods**: This prospective, single-center study includes 91 patients with histologically confirmed RCC. We performed a CEUS within one week prior to nephrectomy. Qualitative parameters (enhancement pattern, heterogeneity, pseudocapsule) and quantitative perfusion metrics were assessed. Logistic regression models were developed to evaluate the diagnostic performance of CEUS in differentiating high-grade (clear cell RCC) from low-grade RCC (papillary and chromophobe). **Results**: Qualitative CEUS findings showed that hyperenhancement and isoenhancement were significantly associated with high-grade RCC (OR = 38.3 and OR = 7.8, respectively; *p* < 0.001 and *p* = 0.014). Hypoenhancement was predominant in low-grade RCC (80.0%). Quantitative parameters, including peak enhancement and wash-in/wash-out area under the curve, significantly differed between tumor grades (*p* < 0.001). A model using qualitative parameters alone achieved an AUC of 0.847 and 81.9% accuracy. Adding quantitative metrics marginally improved performance (AUC 0.912, accuracy 86.2%), though not significantly. **Conclusions**: CEUS provides valuable diagnostic information in differentiating RCC subtypes, with qualitative parameters alone demonstrating strong predictive power. While quantitative analysis slightly enhances diagnostic accuracy, its added value may be limited by technical challenges.

## 1. Introduction

The increasing use of imaging has led to a higher detection rate of focal renal lesions, presenting a significant management challenge for both urologists and radiologists. These lesions can be classified into benign, malignant, and non-tumoral masses (focal pyelonephritis, infectious or inflammatory pseudotumor, etc.). Differentiating benign from malignant solid lesions and between malignant subtypes of solid lesions is crucial in order to choose the correct treatment in every clinical scenario.

Renal cell carcinoma (RCC) is a heterogeneous group of tumors that encompasses different histological and molecular subtypes [1]. Clear cell RCC (ccRCC) is the most common subtype, accounting for 70% of RCC, and is responsible for the most cancer-related deaths among RCC [2,3]. ccRCC is followed in incidence by papillary and chromophobe subtypes, which are less aggressive and have better prognoses, accounting for 10–15% and 5% of renal tumors, respectively, and are considered to be “low-grade malignant renal cell cancers” based on previous publications [2,3].

Evolving multimodal imaging techniques—including ultrasound (US), contrast-enhanced ultrasound (CEUS), computed tomography (CT), and magnetic resonance imaging (MRI)—have significantly advanced diagnostic accuracy, striving to achieve precise identification of histologic subtypes. Over the past decade, this field has been a major focus of research, aiming to enhance diagnostic precision to the highest possible level.

So far, various studies have been published aiming to differentiate renal tumor subtypes by evaluating multiparametric CT and MRI, assessing lesion behavior after contrast administration to derive probability scores [4,5,6]. However, none of these techniques applies quantitative, reproducible parameters, and given the increasing demand for diagnostic accuracy and less invasive approaches in clinical practice, they are insufficiently precise to ensure an accurate and objective assessment on their own.

While CEUS has been extensively studied in cystic renal masses through the Bosniak classification [7,8,9], our study focuses on its application in solid kidney tumors.

The use of contrast-enhanced ultrasound (CEUS) for evaluating solid renal masses is currently limited in clinical practice, primarily to cases involving indeterminate lesions identified on CT or MRI or in patients with contraindications to iodinated or gadolinium-based contrast agents [10]. However, CEUS has shown high sensitivity for detecting lesions with low vascularity profiles [11]. It is also widely used to characterize cystic renal lesions, particularly for identifying solid components within complex cysts. In addition, ultrasound contrast agents allow real-time, continuous assessment of lesion enhancement without radiation exposure and at a lower cost compared to CT and MRI, with broader availability in many clinical settings.

The aim of this study is to analyze perfusion patterns and quantitative parameters to distinguish between different subgroups of RCC using dynamic contrast-enhanced ultrasound.

## 2. Materials and Methods

### 2.1. Study Design and Patient Population

This prospective, single-center study included a cohort of 91 patients who underwent a CEUS over a period of 34 months between November 2016 and September 2019. Eligible patients had a previously diagnosed solid kidney tumor suitable for nephron-sparing surgery or radical nephrectomy. All participants had been diagnosed with a solid kidney tumor based on contrast-enhanced CT. Once surgical treatment was decided, CEUS was performed within one week before surgery, and the histopathological report served as the gold standard reference for our study. Only patients with a final histologic diagnosis of renal cell carcinoma were included.

Patients with lesions identified as benign based on radiological characteristics (e.g., angiomyolipoma) or cystic renal masses were not included in this study. Additionally, 30 patients were excluded due to the inability to achieve accurate perfusion quantification caused by technical software issues.

The final study population comprised 61 patients. Informed consent for the CEUS procedure was obtained from all participants. The study protocol was approved by the local ethics committee and adhered to the principles of the Declaration of Helsinki (1975).

### 2.2. Image Acquisition and Analysis

All contrast-enhanced ultrasound (CEUS) examinations were performed using the same ultrasound system (Acuson S3000, Siemens Healthcare, Erlangen, Germany) equipped with a 3.5–5.5 MHz convex transducer. The CEUS procedures and image analyses were performed by two radiologists with over 15 and 5 years of experience in genitourinary imaging, respectively.

Ultrasound examinations were performed with patients in the supine position, arms raised above the head, and breathing shallowly without restraint.

Once the lesion was identified on grayscale ultrasound, standard CEUS presets using the Cadence™ Contrast Pulse Sequencing (CPS) modality were applied, with a constant wide dynamic range to avoid signal saturation and a low mechanical index (MI < 0.16).

A continuous CEUS cine loop lasting at least 2 min was acquired for each lesion following intravenous administration of a sulfur hexafluoride microbubble contrast agent (2.4 mL, SonoVue, Bracco, Milan, Italy.), followed by a 10 mL saline flush. Image capturing began right after the manual saline flush was finished.

All images were stored in the local picture archiving and communication system (PACS) and were exported to an offline workstation for further analysis.

Tumor characteristics, including size and location, as well as qualitative imaging parameters, were evaluated by the readers. In case of discordance in the lesion qualitative analyses between the two readers, a consensus interpretation was reached.

**Figure 1:**
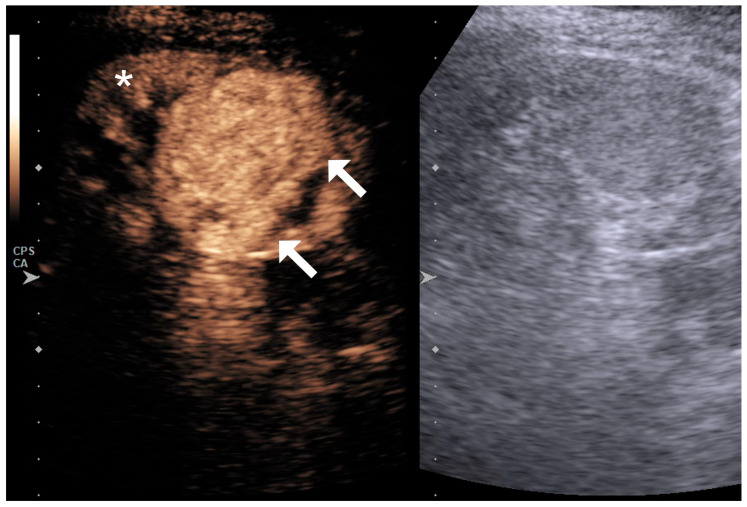
Large endophytic lesion (arrows) that shows hypervascularity compared to the surrounding renal parenchyma (asterisk) on a qualitative assessment.

**Figure 2:**
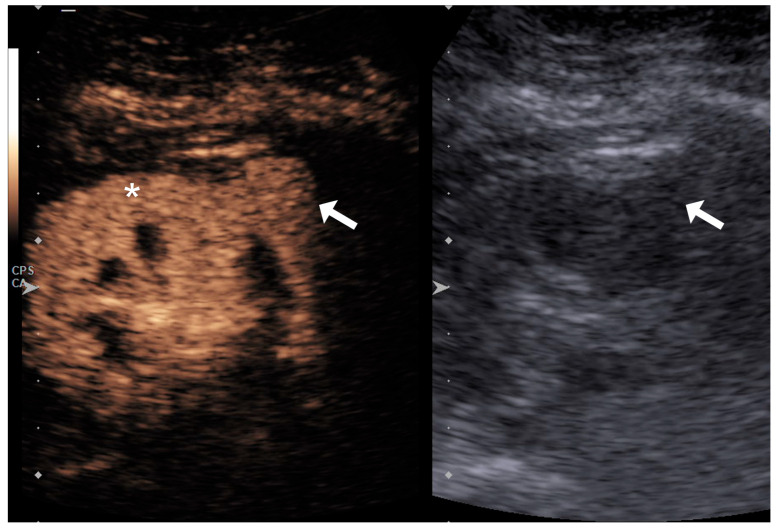
Small exophytic lesion in the lower third of the kidney (arrows) that appears isovascular to the surrounding renal parenchyma (asterisk) on a qualitative assessment.

**Figure 3:**
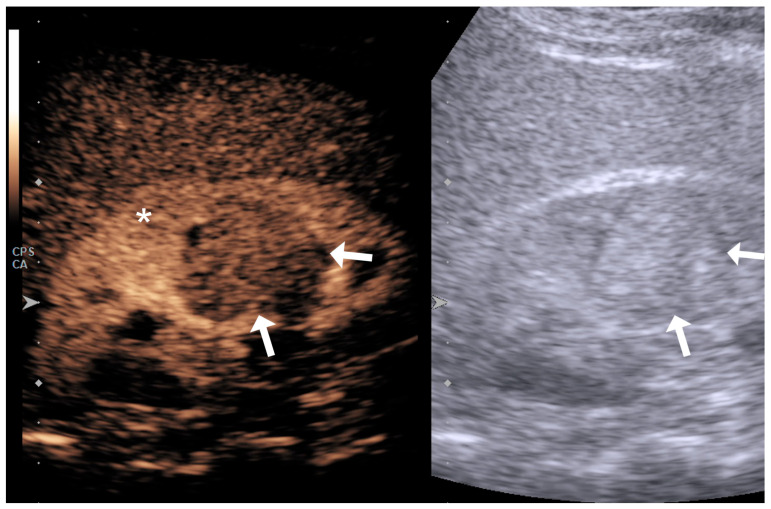
Endophytic lesion with hypovascular appearance (arrows) in comparison to the surrounding renal parenchyma (asterisk) on a qualitative assessment.

The lesion’s enhancement pattern was categorized as hypoenhancing, isoenhancing, or hyperenhancing relative to the adjacent normal renal parenchyma during the phase of homogeneous parenchymal enhancement (Figure 1, Figure 2 and Figure 3).

**Figure 4:**
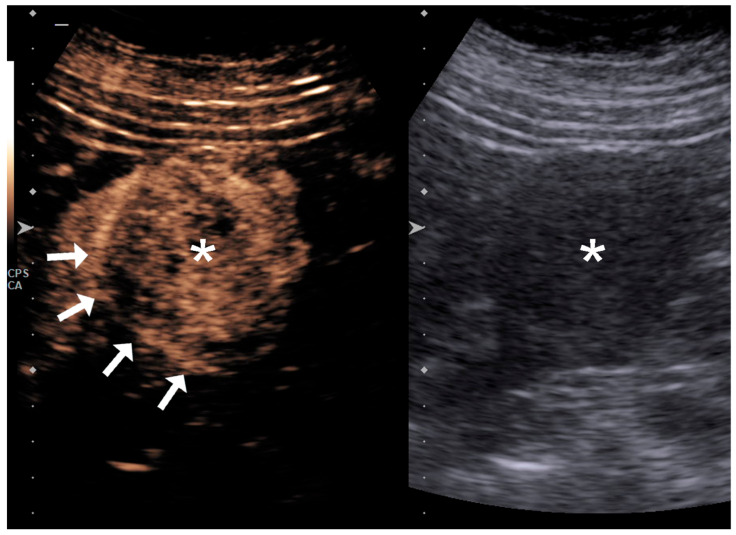
Presence of a pseudocapsule due to tumor growth (asterisk) and compression of the adjacent renal parenchyma with associated fibrous changes (arrows).

Additionally, the presence of heterogeneous enhancement and the appearance of a pseudocapsule—defined as a peripheral rim-like enhancement—were specifically assessed as part of the qualitative analysis (Figure 4).

**Figure 5:**
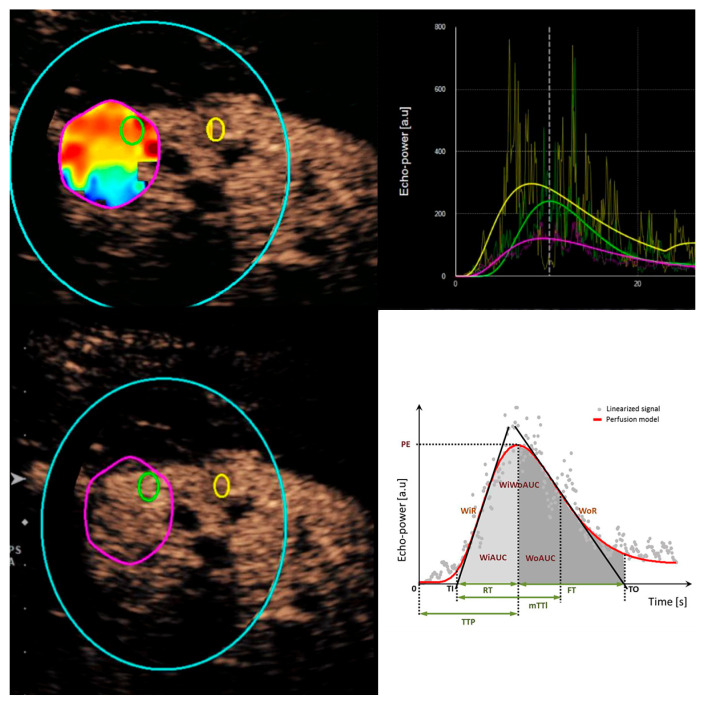
Regions of interest (ROIs) were manually defined. ROI 1 (green): the most hypervascular zone of the lesion, ROI 2 (yellow): the renal cortex adjacent to the lesion, and ROI 3 (fuchsia): encompassing the entire lesion. Perfusion analysis results are displayed as color-coded maps, graphs, and numerical values (not shown). Hyperperfusion appears in red and yellow shades, while hypoperfusion is represented by green and blue shades. The software calculates the following quantitative parameters representing different segments of the time–intensity curve (TIC): Peak Enhancement (PE), Wash-in Area Under the Curve (WiAUC), Rise Time (RT), Mean Transit Time local (mTTl), Time To Peak (TTP), Wash-in Rate (WiR), Wash-in Perfusion Index (WiPI), Wash-out Area Under the Curve (WoAUC), Wash-in and Wash-out AUC (WiWoAUC), Fall Time (FT), Wash-out Rate (WoR), Quality of Fit (QOF), and ROI area (Area, in cm^2^) (see Supplementary Material).

A quantitative perfusion pattern analysis was conducted using an external quantification tool (VueBox v.6.2, Bracco, Italy), a dedicated software for dynamic CEUS studies that provides color-coded visualizations and integrated motion correction. Regions of interest (ROIs) were manually defined in three specific areas of the image: ROI 1 (green) in the most hypervascular zone of the lesion, ROI 2 (yellow) in the renal cortex adjacent to the lesion, and ROI 3 (fuchsia) encompassing the entire lesion (Figure 5). The sizes of the ROIs for the hypervascular part of the lesion and the normal parenchyma (ROI 1 and ROI 2) were standardized across all cases, ensuring minimal variations. 

### 2.3. Statistical Analyses

Statistical analyses were conducted using IBM SPSS Statistics 26.0. Categorical variables were expressed as frequencies and percentages, with group differences assessed using Pearson’s chi-square test or Fisher’s exact test for 2 × 2 tables. Continuous variables were reported as medians and interquartile ranges (IQRs) and compared using the Mann–Whitney U test. A binary logistic regression model was employed to identify independent predictors of renal tumor characteristics based on contrast-enhanced ultrasound (CEUS) findings. Variables with potential significance in univariate analysis were included in the multivariate model. The regression analysis considered odds ratios (ORs) with 95% confidence intervals (CIs), ensuring minimization of multicollinearity through variance inflation factor assessment. Model performance was evaluated using the Hosmer–Lemeshow goodness-of-fit test, and significance was set at α = 0.05.

## 3. Results

### 3.1. Univariate Analysis

**Figure 6:**
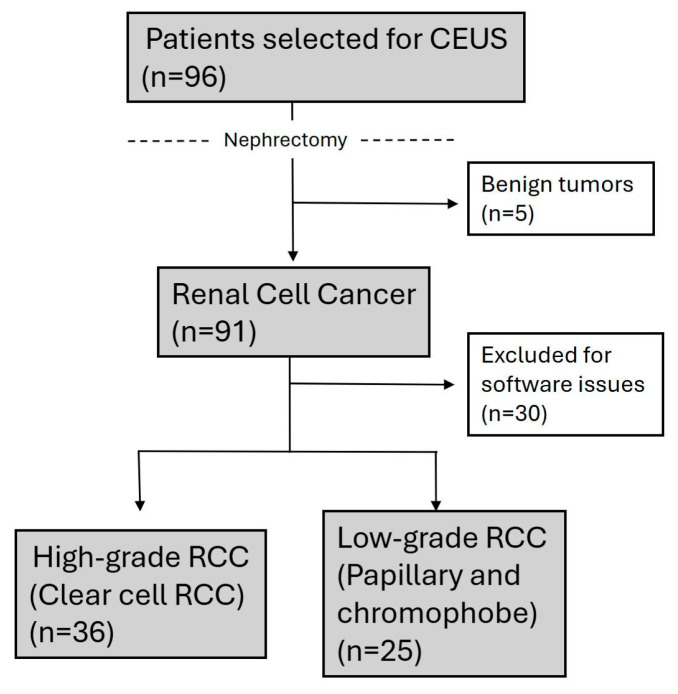
Flowchart of patient selection and study design.

The final post-surgical histologic analyses demonstrated a sample consisting of 25 low-grade RCC cases (18 papillary RCC and 7 chromophobe RCC), along with 36 high-grade RCC cases (clear cell RCC). Additionally, five oncocytomas were identified (Figure 6). Differences in demographic and clinical features, including age (median: 65 vs. 60 years; *p* = 0.159) and male predominance (68.0% vs. 77.8%; *p* = 0.393), were not statistically significant. Similarly, neither tumor size (median: 3.60 cm vs. 5.45 cm; *p* = 0.129), tumor location (e.g., superior pole: 32.0% vs. 22.2%; *p* = 0.535), nor exophytic growth (76.0% vs. 69.4%; *p* = 0.574) showed significant differences (Table 1).

Evaluation of qualitative CEUS parameters revealed significant differences in heterogeneity (48.0% vs. 83.3%; *p* = 0.003) and enhancement patterns between low-grade RCC (chromophobe and papillary) and high-grade RCC (clear cell). Hypoenhancement was predominant in low-grade RCC (80.0%) and significantly less frequent in high-grade RCC (16.7%; *p* < 0.001). Conversely, hyperenhancement was more common in high-grade RCC (63.9%) compared to low-grade RCC (8.0%) (Table 2).

Quantitative CEUS parameters, such as PE1 (*p* = 0.001), WiAUC1 (*p* < 0.001), and WoAUC1 (*p* < 0.001), differed significantly between the two groups. Other metrics, including WiPI1 (*p* < 0.001) and WiR1 (*p* = 0.009), also showed substantial variations (Table 3).

Parameters representing different segments of the time–intensity curve (TIC) are shown in Table 3: Peak Enhancement (PE), Wash-in Area Under the Curve (WiAUC), Rise Time (RT), Mean Transit Time local (mTTl), Time To Peak (TTP), Wash-in Rate (WiR), Wash-in Perfusion Index (WiPI), Wash-out Area Under the Curve (WoAUC), Wash-in and Wash-out AUC (WiWoAUC), Fall Time (FT), Wash-out Rate (WoR), Quality of Fit (QOF), and ROI area (Area, in cm^2^) (see Supplementary Material).

### 3.2. Binary Logistic Regression Model

The final binary logistic regression model incorporated both qualitative and quantitative CEUS parameters to estimate the probability of a tumor being classified as high-grade RCC. A block-wise logistic regression approach was used to evaluate the added predictive value of quantitative CEUS parameters over qualitative CEUS parameters, such as CEUS heterogeneity and enhancement pattern. This multivariate analysis aimed to identify which imaging features and CEUS parameters significantly increase the likelihood of a tumor being high-grade RCC. For the model that includes qualitative parameters alone, CEUS enhancement showed a strong impact: isoenhancement increased the odds of high-grade RCC by 7.78 times (OR = 7.778, 95% CI: 1.522–39.754, *p* = 0.014), and hyperenhancement increased it by 38.33 times (OR = 38.333, 95% CI: 6.941–211.694, *p* < 0.001) compared to a hypovascular pattern. The model’s AUC was 0.847 (95% CI: 0.744–0.950), with an overall accuracy of 81.9% at the optimal probability cut-off of 0.465 (Table 4). Although heterogeneous enhancement was significant in the univariate analysis (*p* = 0.003), it was not included in the logistic regression model (*p* = 0.164), likely due to collinearity with CEUS enhancement patterns. The model prioritized stronger independent predictors, suggesting that heterogeneous enhancement alone does not independently differentiate tumor grade. We also applied regularization techniques such as LASSO regression to address multicollinearity; however, this approach did not enhance model performance or variable selection, further supporting the limited independent contribution of heterogeneous enhancement.

Adding quantitative parameters that were significant in the bivariate analysis (e.g., logWoAUC1) slightly improved the model’s performance, with an AUC of 0.912 (95% CI: 0.836–0.988) and an accuracy of 86.2% (Table 5). However, Hanley and McNeil’s test indicated no statistically significant difference between the two models’ AUCs (z = 1.10, p > 0.05). Consequently, qualitative CEUS parameters alone provided robust diagnostic performance. While the second model that included quantitative parameters demonstrated a slight improvement in specificity (91.7% vs. 80.0%) and Youden Index (0.741 vs. 0.633), the differences were not statistically significant, suggesting that qualitative CEUS parameters are sufficient for accurate differentiation of RCC subtypes.

## 4. Discussion

This study explored the utility of contrast-enhanced ultrasound (CEUS) in distinguishing between high-grade and low-grade renal cell carcinoma (RCC), with an emphasis on both qualitative and quantitative parameters. The findings highlight the potential of CEUS as a diagnostic tool and corroborate some previously published studies [12,13,14,15,16], particularly through qualitative enhancement patterns and selected quantitative metrics, while acknowledging certain limitations due to sample size and the complexity of multivariate modeling.

### 4.1. Qualitative Evaluation

In this study, we observed significant differences in the qualitative CEUS parameters between low- and high-grade RCC. Our evaluation revealed that CEUS enhancement patterns substantially impact the likelihood of high-grade RCC. Specifically, isoenhancement increased the odds of high-grade RCC by 7.78 times (OR = 7.778, 95% CI: 1.522–39.754, *p* = 0.014), while hyperenhancement raised this probability by 38.33 times (OR = 38.333, 95% CI: 6.941–211.694, *p* < 0.001) compared to hypoenhancement. These findings reinforce the role of CEUS qualitative parameters in tumor characterization and their potential utility in risk stratification, corroborating findings in previous studies [17,18,19,20]. Zhu et al. reported that in small renal tumors, hyperenhancement during the arterial phase was consistently linked to malignancy, whereas isoenhancement was exclusively associated with benign lesions [18]. Other authors have described that, among the qualitative parameters, a rapid and inhomogeneous wash-in pattern is highly accurate in distinguishing malignant lesions from benign tumors [19]. However, these studies did not assess or compare enhancement patterns across different malignant tumor subtypes. In comparison, Liang et al. noted that a hypervascular pattern is more frequently observed in low-grade than in high-grade clear cell renal cell carcinomas, particularly in small tumors [16]. It is important to mention that their analysis focused exclusively on comparing different histological grades within clear cell RCC, whereas our study evaluated CEUS enhancement patterns across different grades of RCC encompassing multiple histological subtypes.

Zhu et al. also highlighted the role of CEUS in assessing tumor heterogeneity and the presence of a pseudocapsule as distinguishing features of renal tumor types, particularly varying by size [18]. In our study, tumor heterogeneity was also significantly higher in high-grade RCC in the univariate analysis (*p* = 0.003); however, it was not included in the logistic regression model (*p* = 0.164). This suggests that heterogeneous enhancement is more frequent in high-grade RCC, as it has been described previously using CT and MRI [21,22,23]. However, it was not an independent predictor alone of tumor grade in our study.

Tufano et al. found that heterogeneous enhancement and rim-like enhancement were strong predictors of malignancy, with an AUC of 82.5% for heterogeneous enhancement in differentiating clear cell RCC from oncocytoma [19]. Other studies also detected a correlation with the presence of a pseudocapsule [20,24,25]. In contrast, we did not observe any association between rim-like enhancement and tumor grade in our cohort. However, it is important to note that, unlike other studies, we excluded oncocytomas from our analysis, which may have influenced the differences in enhancement pattern findings.

Our results, including qualitative parameters, align with other studies emphasizing the diagnostic reliability of rapid wash-in patterns in CEUS and tumor heterogeneity for high-grade malignant lesions but not the presence of a pseudocapsule sign [20]. These comparisons underscore the robustness of CEUS as a diagnostic tool for characterizing renal tumors, particularly in distinguishing malignancy grades through enhancement patterns.

The diagnostic model based solely on qualitative parameters achieved an AUC of 0.847 with 81.9% accuracy, underscoring the utility of qualitative CEUS in clinical practice. These results suggest that qualitative assessments can serve as a reliable, resource-efficient approach for characterizing renal masses.

### 4.2. Quantitative Evaluation

The quantitative analysis of perfusion parameters offered slight incremental improvements in diagnostic performance. Metrics such as Peak Enhancement (PE1), Wash-in Area Under the Curve (WiAUC1), and relative enhancement percentage (PE1-PE2/100), which are related to tumor hypervascularization, and Wash-out Area Under the Curve (WoAUC1) showed significant differences between tumor subtypes. Incorporating these parameters into the diagnostic model raised the AUC to 0.912 and improved accuracy to 86.2%. However, the added complexity of quantitative analysis must be balanced against its marginal gains in diagnostic accuracy. The AUC of the second block is higher (0.912 vs. 0.847), indicating a slightly better diagnostic performance when quantitative parameters are included; however, the difference between the two AUCs is not statistically significant at the 5% or even the 10% level. This means that while the added quantitative parameters provide a slight numerical improvement in diagnostic accuracy, they do not significantly enhance the model’s diagnostic ability beyond what the qualitative parameters already achieve. Previous studies where quantitative CEUS parameters were analyzed [13,14,15,16,20,24,25] showed that parameters like peak intensity and area under the curve (AUC) displayed high sensitivity (94–99%) and specificity (92%) for distinguishing malignancies [20]. Other authors confirm that CEUS can differentiate clear cell RCC (ccRCC) from non-clear cell RCC (nccRCC) with high sensitivity (99.5%) and has a comparable diagnostic performance to contrast-enhanced CT [24]. In this study, CEUS parameters, including arrival time between tumor and cortex (∆ATtumor-cortex) and time to peak of the tumor (TTPtumor), provide significant differentiation between ccRCC and nccRCC [24]. Dipinto also highlights CEUS as a valuable tool for distinguishing RCCs from AMLs, particularly fat-poor AMLs, by evaluating enhancement patterns, pseudocapsule signs, wash-in/wash-out times, and key quantitative parameters such as time-to-peak (TTP), mean transit time (mTT), tumor-to-cortex enhancement ratio (TOC), maximum intensity (IMAX), and area under the curve (AUC) [15].

Notably, in our study, the qualitative approach alone demonstrated strong diagnostic performance in differentiating low-grade from high-grade RCC, questioning the necessity of quantitative analysis in routine clinical settings. This insight could streamline the integration of CEUS into standard workflows, reducing reliance on specialized tools and extensive post-processing.

Despite the potential benefits, our study highlighted key limitations of quantitative evaluation. Technical challenges, including motion artifacts, difficulties in continuous visualization, and software-related errors, resulted in the exclusion of 31% of patients from the final analysis. These obstacles underscore the need for more robust and user-friendly quantitative analysis tools. The strong performance of qualitative CEUS, combined with its accessibility and safety, positions it as a valuable diagnostic tool in clinical practice. While overcoming the current challenges of quantitative analysis may enhance its utility, it is also possible that qualitative assessment alone may be sufficient in most cases.

The dynamic, real-time nature of CEUS provides distinct advantages over traditional imaging modalities like CT and MRI. Our findings reinforce its utility, particularly in patients with contraindications to iodinated or gadolinium-based contrast agents. CEUS also enables continuous observation of enhancement patterns for qualitative analyses, which proved critical for distinguishing tumor subtypes in our cohort.

### 4.3. Limitations and Challenges

Although the findings are promising, CEUS has several limitations, and this study encountered some of these challenges, particularly in CEUS quantification. According to the published literature and based on our experience, these difficulties can be grouped into four main categories [11,12,13,16,19,20,26].

First, patient-related factors influenced image quality and data acquisition. Variability in the acoustic window, determined by patient anatomy and body habitus, affected image clarity. Additionally, inconsistent breath-holding among patients led to fluctuations in CEUS signal stability. Hemodynamic factors, such as differences in cardiovascular status and cardiac output, also impacted contrast distribution and wash-out rates.

Second, lesion-related factors posed further challenges. Tumor location played a critical role, as deeper or posteriorly positioned lesions were harder to evaluate due to reduced ultrasound penetration. Intratumoral heterogeneity complicated the placement of the region of interest (ROI) and affected quantification accuracy. Furthermore, smaller lesions were more difficult to assess precisely, potentially influencing CEUS parameter measurements.

Third, CEUS-related factors contributed to variability in the procedure. The inconsistent stability and dispersion of microbubbles can impact enhancement uniformity. Furthermore, variations in injection techniques, as well as differences in dose and injection velocity, could influence perfusion dynamics and the final quantification parameters.

Finally, quantification-related factors can also impact the accuracy and reproducibility of CEUS analysis in general. The type of data used, whether DICOM or raw (RAW) data, may play a significant role, as raw data offers higher precision but is not always available in routine clinical practice. In our study, we used DICOM files, which are more widely accessible but may offer lower precision than raw data. Moreover, different post-processing software solutions for CEUS quantification can introduce variability in parameter extraction, further complicating standardization efforts.

These limitations underscore the need for improved standardization in CEUS acquisition, processing, and interpretation. Future research should focus on optimizing protocols and refining post-processing algorithms to enhance reproducibility and clinical applicability.

Furthermore, our study did not include benign lesions, and therefore, it cannot assess the ability to differentiate between benign and malignant lesions, which remains an important area for future research. We also did not evaluate histologic grade within subgroups or correlate CEUS findings with other pathological features, such as necrosis or microvascular invasion, due to inconsistent reporting and small sample sizes across subtypes. Additionally, multicenter studies with larger sample sizes are needed to validate our findings and establish definitive guidelines for the use of CEUS in renal tumor evaluation.

## 5. Conclusions

Our study underscores the diagnostic value of contrast-enhanced ultrasound (CEUS) in distinguishing between low-grade and high-grade kidney cancers, with qualitative assessments demonstrating strong and reliable performance. While quantitative analysis offered additional insights, it contributed minimally to diagnostic discrimination in our cohort and was limited by technical and operational challenges. Addressing these limitations may enhance its future utility; however, it is also possible that quantitative approaches may provide only marginal added value in this setting. Future work should focus on consolidating the strengths of qualitative evaluation while carefully exploring the potential and limits of quantitative methods in routine clinical practice.

## Figures and Tables

**Table 1:** Comparison of demographic and tumor characteristics between groups.

Variable	RCC Low Grade (n = 25)	RCC High Grade (n = 36)	*p* Value
Age (median, IQR)	65.00 (56.00, 74.00)	60.00 (51.50, 66.00)	0.159
Male Sex (N, %)	17 (68.0%)	28 (77.8%)	0.393
Localization (N, %)			0.535
Superior	8 (32.0%)	8 (22.2%)	
Medium	6 (24.0%)	13 (36.1%)	
Inferior	11 (44.0%)	15 (41.7%)	
Exophytic (N, %)	19 (76.0%)	25 (69.4%)	0.574
Size (median, IQR)	3.60 (2.50, 6.50)	5.45 (3.90, 6.15)	0.129

*p*-values corresponding to chi-squared tests for categorical parameters and Mann–Whitney U tests for continuous parameters.

**Table 2:** Comparison of qualitative CEUS parameters between low- and high-grade RCC.

Variable	RCC Low Grade (n = 25)	RCC High Grade (n = 36)	*p* Value
Heterogeneous (N,%)	12 (48.0%)	30 (83.3%)	0.003 **
Enhancement (N,%)	3 (12.0%)		
Hypo	20 (80.0%)	6 (16.7%)	0.000 **
Iso	3 (12.0%)	7 (19.4%)	0.505
Hyper	2 (8.0%)	23 (63.9%)	0.000 **
Pseudocapsule sign (N,%)	6 (24.0%)	14 (38.9%)	0.223

*p*-values corresponding to chi-squared tests for categorical parameters and Mann-Whitney tests for continuous parameters: ** *p*-value < 0.01.

**Table 3:** Comparison of quantitative CEUS parameters between low- and high-grade RCC.

Quantitative Parameters (Median, IR)	RCC Low Grade (n = 25)	RCC High Grade (n = 36)	*p* Value
PE1	4.83 × 10^7^ (1.77 × 10^7^, 8.41 × 10^7^)	1.69 × 10^8^ (6.90 × 10^7^, 5.37 × 10^8^)	0.001 **
WiAUC1	1.56 × 10^8^ (6.19 × 10^7^, 2.88 × 10^8^)	7.71 × 10^8^ (3.01 × 10^8^, 2.23 × 10^9^)	0.000 **
RT1	5561.50 (4070.50, 7768.50)	7141.00 (5448.00, 9912.00)	0.052
mTTl1	23,277.00 (13,674.00, 46,630.00)	27,244.00 (17,809.00, 46,651.00)	0.564
TTP1	9161.00 (7362.00, 11,124.00)	12,044.00 (8361.00, 14,223.00)	0.086
WiR1	1.04 × 10^7^ (5.46 × 10^6^, 2.05 × 10^7^)	5.79 × 10^7^ (9.05 × 10^6^, 1.65 × 10^8^)	0.009 **
WiPI1	3.06 × 10^7^ (1.13 × 10^7^, 5.38 × 10^7^)	1.03 × 10^8^ (4.44 × 10^7^, 3.36 × 10^8^)	0.000 **
WoAUC1	2.77 × 10^8^ (9.73 × 10^7^, 4.54 × 10^8^)	1.74 × 10^9^ (6.53 × 10^8^, 3.45 × 10^9^)	0.000 **
WiWoAUC1	4.33 × 10^8^ (1.63 × 10^8^, 7.20 × 10^8^)	2.35 × 10^9^ (9.25 × 10^8^, 5.62 × 10^9^)	0.000 **
FT1	10,473.00 (9674.00, 19,684.00)	14,608.00 (11,801.00, 22,050.00)	0.077
WoR1	4.78 × 10^6^ (2.52 × 10^6^, 8.06 × 10^6^)	2.35 × 10^7^ (3.06 × 10^6^, 5.57 × 10^7^)	0.040 *
QOF1	66,164.50 (31,111.00, 85,726.00)	74,451.00 (54,181.00, 83,754.00)	0.483
Area1	0.18 (0.13, 0.30)	0.25 (0.14, 0.37)	0.257
PE3	3.07 × 10^7^ (9.90 × 10^6^, 6.68 × 10^7^)	7.60 × 10^7^ (3.73 × 10^7^, 2.09 × 10^8^)	0.001 **
WiAUC3	1.23 × 10^8^ (4.30 × 10^7^, 2.84 × 10^8^)	3.95 × 10^8^ (1.82 × 10^8^, 1.23 × 10^9^)	0.005 **
RT3	7059.00 (5215.00, 8917.00)	7814.00 (5549.00, 11,651.00)	0.380
MTTl3	24,305.00 (13,870.00, 51,630.00)	37,267.00 (21,873.00, 100,282.00)	0.253
TTP3	6.20 × 10^6^ (2.44 × 10^6^, 1.11 × 10^7^)	2.21 × 10^7^ (5.26 × 10^6^, 4.66 × 10^7^)	0.008 **
WiR3	1.95 × 10^7^ (6.38 × 10^6^, 4.21 × 10^7^)	4.80 × 10^7^ (2.55 × 10^7^, 1.34 × 10^8^)	0.001 **
WiPI3	1.85 × 10^8^ (4.93 × 10^7^, 4.16 × 10^8^)	6.85 × 10^8^ (2.85 × 10^8^, 2.15 × 10^9^)	0.004 **
WoAUC3	3.33 × 10^8^ (7.95 × 10^7^, 7.57 × 10^8^)	1.12 × 10^9^ (5.40 × 10^8^, 3.45 × 10^9^)	0.002 **
WiWoAUC3	14,010.00 (10,619.00, 19,822.00)	15,360.00 (10,163.00, 21,718.00)	0.708
FT3	2.45 × 10^6^ (1.14 × 10^6^, 4.27 × 10^6^)	7.20 × 10^6^ (1.85 × 10^6^, 1.61 × 10^7^)	0.005 **
WoR3	2.48 × 10^6^ (1.69 × 10^6^, 4.88 × 10^6^)	8.09 × 10^6^ (2.21 × 10^6^, 1.62 × 10^7^)	0.014 *
QOF3	8.62 × 10^4^ (5.73 × 10^4^, 9.61 × 10^4^)	9.01 × 10^4^ (6.84 × 10^4^, 9.54 × 10^4^)	0.752
Area3	0.007 (0.004, 0.011)	0.010 (0.007, 0.015)	0.036 *
(PE1-PE2)/100	14.77 (−85.46, 91.05)	107.72 (13.87, 315.71)	0.002 **

*p*-values corresponding to chi-squared tests for categorical parameters and Mann-Whitney tests for continuous parameters: * *p*-value < 0.05, ** *p*-value < 0.01.

**Table 4:** Binary logistic regression analysis of CEUS parameters for predicting high-grade RCC.

Variable	B	S.E.	Wald	df	Sig.	Exp(B)	95% CI for Exp(B)
CEUS enhancement: Hypo (ref.)			19	2	0		
CEUS enhancement: Iso	2.051	0.832	6.073	1	0.014	7.778	[1.522, 39.754]
CEUS enhancement: Hyper	3.646	0.872	17.491	1	0.000	38.333	[6.941, 211.694]
Constant	−1.204	0.465	6.690	1	0.010	0.300	
Cut-off Probability	Sensitivity	Specificity	Youden Index	Total Accuracy			
*p* = 0.465	83.3%	80.0%	0.633	81.9%			

Exp (b) coefficients < 1 indicate a reduced probability of high-grade RCC. Exp (b) coefficients > 1 indicate an increased probability of high-grade RCC. ref.: reference category. B: Beta Coefficient, S.E.: Standard Error, Wald: Wald Statistic, df: Degrees of Freedom, Sig.: Significance, Exp (B): Odds Ratio, 95% CI for Exp (B): 95% Confidence Interval for the Odds Ratio.

**Table 5:** Logistic regression model combining qualitative and quantitative CEUS parameters for predicting high-grade RCC.

Variable	B	S.E.	Wald	df	Sig.	Exp (B)	95% CI for Exp (B)
CEUS heterogeneous: Yes	1.827	0.966	3.579	1	0.059	6.214	[0.936, 41.245]
CEUS enhancement: Hypo (ref.)			9.391	2	0.009		
CEUS enhancement: Iso (ref.)	1.902	1.124	2.862	1	0.091	6.697	[0.740, 60.615]
CEUS enhancement: Hyper	3.209	1.057	9.214	1	0.002	24.747	[3.117, 196.475]
logWoAUC1	1.384	0.690	4.019	1	0.045	3.989	[1.031, 15.431]
Constant	−14.682	6.284	5.459	1	0.019	0.000042	
Cut-off Probability	Sensitivity	Specificity	Youden Index	Total Accuracy			
*p* = 0.649	82.4%	91.7%	0.741	86.2%			

B: Beta Coefficient, S.E.: Standard Error, Wald: Wald Statistic, df: Degrees of Freedom, Sig.: Significance, Exp(B): Odds Ratio, 95% C.I. for Exp(B): 95% Confidence Interval for the Odds Ratio.

## Data Availability

The data presented in this study are available from the corresponding authors upon request.

## Supplementary Materials

The following supporting information can be downloaded at: https://www.mdpi.com/article/10.3390/diagnostics15141795/s1, Supplementary Material file: Time-Intensity-Curve (TIC) parameters in CEUS.

## Abbreviations

The following abbreviations are used in this manuscript:

CEUS	Contrast-Enhanced Ultrasound
RCC	Renal Cell Carcinoma
ccRCC	Clear Cell Renal Cell Carcinoma
CT	Computed Tomography
MRI	Magnetic Resonance Imaging
US	Ultrasound
