# Supplementary material for: Qualitative and Quantitative Analysis of Contrast-Enhanced Ultrasound in the Characterization of Kidney Cancer Subtypes
Source: Diagnostics (Basel). 2025 Jul 16;15(14):1795. doi: 10.3390/diagnostics15141795 (PMC12293668; doi:10.3390/diagnostics15141795)
Supplement: Supplementary file 1 [file diagnostics-15-01795-s001.zip › diagnostics-3736792-supplementary.pdf]

Supplementary Material

Explanation of each parameter representing different segments of a Time-Intensity Curve (TIC) in Contrast-Enhanced Ultrasound (CEUS):

Peak Enhancement (PE)
The maximum intensity reached in the TIC; reflects the highest contrast concentration in the region of interest (ROI), indicating peak perfusion.

Wash-in Area Under the Curve (WiAUC)
The area under the curve during the contrast inflow phase; represents the total amount of contrast agent delivered to the ROI during wash-in.

Rise Time (RT)
Time from the beginning of the wash-in to the peak intensity; indicates how quickly the contrast reaches its peak, often related to perfusion velocity.

Mean Transit Time local (mTTl)
Average time the contrast remains within the ROI; reflects both inflow and outflow dynamics, useful for assessing perfusion efficiency.

Time To Peak (TTP)
Time from the arrival of contrast to the point of peak enhancement; includes both the arrival and accumulation phases, useful for comparing tissue perfusion timing.

Wash-in Rate (WiR)
The slope of the ascending (wash-in) part of the TIC; indicates the rate at which contrast enters the ROI, related to blood flow velocity.

Wash-in Perfusion Index (WiPI)
Ratio of WiAUC to Rise Time; a composite index representing both the amount and speed of contrast inflow, helpful in distinguishing tissue types.

Wash-out Area Under the Curve (WoAUC)
The area under the curve during the wash-out phase; reflects the total contrast volume leaving the ROI over time.

Wash-in and Wash-out AUC (WiWoAUC)
The total area under the TIC including both inflow and outflow phases; represents the overall perfusion through the ROI during the entire observation.

Fall Time (FT)
Time from peak intensity to the return to baseline during wash-out; measures how long the contrast stays in the tissue before clearing.

Wash-out Rate (WoR)
The slope of the descending (wash-out) segment of the TIC; reflects how rapidly the contrast exits the ROI.

Quality of Fit (QOF)
A statistical measure of how well the TIC data fits the chosen model (e.g., gamma-variate); higher values indicate a more reliable curve analysis.

ROI Area (Area, in cm^2)
The physical size of the region of interest used for TIC generation; important for comparing perfusion metrics across lesions or tissues.
